# Representative Characteristics of the HIV/AIDS-infected Young Persons in Romania in Terms of Their Personal and Professional Values, Needs and Resources

**Published:** 2017-08

**Authors:** Felicia ANDRIONI, Ion PETRICĂ

**Affiliations:** 1. Dept. of Socio-Human Sciences, Faculty of Sciences, University of Petroşani, Petroşani, Romania; 2. Faculty of Social Sciences, ”Eftimie Murgu” University of Reşita, Reşita, Romania

## Dear Editor-in-Chief

The HIV/AIDS pandemic has tremendously spread at a global level with serious implications upon population. Young population is mainly affected from the point of view of education, family, social and professional level, and the pandemic’s effects on the young individuals stir concern. Romania reported 1094 cases of AIDS in children, representing half of the total number of paediatric AIDS in Europe in 1990 (1). At present, under such global circumstances, the HIV/AIDS-affected young persons in Romania display a series of special traits too, in terms of psychological, social, and professional development, especially worth considering when observing that the HIV/AIDS-affected young persons (19–29 years old) represent a majority as compared with the rest of the HIV-infected population (2). Romania registers more frequent adult infections together with the spread of the infection among those population groups adopted risky sexual behaviours and among those who use injected drugs; the future prognosis of such groups is not a favourable one.

The object of this investigation targeted the designing of an up-dated profile of the HIV/AIDS-affected young persons in terms of their personal and professional needs, interests, resources and values. The pragmatic-applicative research was carried out by the Service for Information, Counselling, and Professional Guidance (SICPG) of the Jiu Valley region, and display quantitative data relevant for accomplishing the objective of our research.

Four research questions were followed in this study and the investigated population includes 100 young persons in the Jiu Valley, affected by the human immune-deficiency virus; their age ranges between 19 and 40 yr old, and a sex distribution of 56% males and 44% females. The distribution of the subjects according to age is as follows: 77% are between 19 and 25 years old, 23% are between 26 and 40 yr old. The research methodology involves the use of the enquiry relying on a interests/values self-administered questionnaire (3) answered by the young persons affected by the human immune-deficiency virus. In our case, the most relevant means of analysis was the inventory of interests, needs, and resources, owing to the fact that it allows self-administration and, implicitly, self-knowledge, being the most frequently used means of guidance intervention. The data are further analysed and interpreted descriptively in accordance with the answers given by the HIV-affected respondents in the Jiu Valley. As far as the first analysis element is concerned - the inventory of the respondents’ values, the respondents considered that the most important values (in a descendant order of their importance) are the following ones: helping other individuals, job independence/autonomy, moral fulfilment, friendship and collegiality, artistic creativity, safety, social status, and affiliation/recognition as a member of a labour group. A series of conclusive aspects can be asserted in connection with the second analysis element - the inventory of the vocational needs of the respondents show us that in terms of the degree of participation of the young people in career counselling activities, 75 % of the investigated persons said that they did not participate in such activities; meanwhile, most (67%) respondents asserted that they had no idea about a future career; the rest of them considered that they were aware of the career they are going to follow. These data show that the need for career counselling is a real issue for these young persons.

In terms of the need for assistance in choosing a career, the following needs were registered: the need for professional information (88%); for exploring the interests, abilities (88%); for professional guidance (85%); for self-esteem (89%); for inter-personal valorisation (85%); for social utility (61%). Let us notice that all the types of needs enumerated display very high values because of the subjects’ answers, who considered they need assistance in choosing their career that regards all the components previously mentioned. As far as the resources owned by the subjects at the moment the questionnaire was administered are concerned, the following categories of resources were enumerated: family (26%), group of friends (90%), owning a house (12%), money (56%), wealth (12%), owning a car (17%), education-diploma (74%), competence in a certain domain (52 %), employment (17%), a pleasant job (52%), others (36%). In the case of HIV/AIDS-affected young persons, their personal system of values reflects their behaviour habit as well as the decisions all persons would take under various daily circumstances (4), and the investigation of the career-relevant personal traits of the HIV-affected young people relies on the knowledge and self-knowledge of their own interests, values, needs, resources, personality traits, aptitudes, and habits (5). In spite of their illness, the HIV/AIDS-infected young people pay an increased attention to their preoccupations that regard their career and employment, namely to those occupational and vocational interests which are directly correlated with their financial interests; the investigated subjects also pay attention to their social/locative interests and to their family interests.

The HIV-affected young persons are aware of the importance of the domain of professional interest, displaying accurately the professional options they would like to explore.
